# Transcatheter Aortic Valve Replacement in Systemic Sclerosis: A Case Series and Review of Short- and Long-Term Outcomes

**DOI:** 10.31138/mjr.280525.ehr

**Published:** 2026-01-28

**Authors:** Chris T. Derk, Ahmadreza Badali, Esra Mehmetoglu, Anvitha Mummadisetty, Amit Syal, Konstantinos Parperis

**Affiliations:** 1 Department of Internal Medicine, Division of Rheumatology, University of Pennsylvania, Philadelphia, USA;; 2 Tabriz University of Medical Sciences, Tabriz, Iran;; 3 Istanbul Faculty of Medicine, Istanbul, Turkey;; 4 Kakatiya Medical College, Warangal, India;; 5 Sidney Kimmel Medical College at Thomas Jefferson University, Philadelphia, PA, USA;; 6 Department of Internal Medicine, Division of Rheumatology, University of Cyprus Medical School, Nicosia, Cyprus

**Keywords:** aortic valve stenosis, autoimmune diseases, echocardiography, retrospective studies, systemic sclerosis, transcatheter aortic valve replacement

## Abstract

**Objectives:**

A higher frequency of valvular heart disease is seen among Systemic Sclerosis (SSc) patients. Advanced aortic valve stenosis leads to significant morbidity in these patients with multiple other comorbidities. In this study, we aim to define the short- and long-term outcomes of transcatheter aortic valve replacement (TAVR) procedures in SSc patients.

**Methods:**

We undertook a retrospective chart review of all patients with SSc who underwent a TAVR procedure at our institution over a defined 11-year period. Demographics as well as short- and long-term outcomes were identified.

**Results:**

Fourteen SSc patients underwent a TAVR procedure between 2012 and 2023. They were predominantly older Caucasian female patients with limited cutaneous SSc (lcSSc) with advanced aortic stenosis. Only one patient had a readmission within 30 days due to post-op heart failure and subsequently had to have the TAVR reversed to a SAVR.

**Conclusions:**

TAVR is a well-tolerated procedure in SSc patients with advanced aortic stenosis and multiple comorbidities.

## INTRODUCTION

Systemic sclerosis (SSc) is an autoimmune rheumatic disease characterised by vasculopathy, autoimmunity, and tissue fibrosis. Significant cardiovascular complications involve the myocardium, the pericardium, the conduction system and the heart valves.^1–3^ Although valvular heart disease (VHD) is not a common manifestation of SSc, studies indicate a higher frequency of VHD among SSc patients, ^4–7^ which correlates with poorer survival rates.^8^

Screening for valvular disease in asymptomatic patients is essential for early diagnosis and better management. Aortic valve involvement in SSc manifests as aortic valve stenosis (AS), which is the narrowing of the aortic valve opening due to calcification and fibrosis.^9^ Studies have shown that patients with SSc exhibit increased aortic stiffness, and a higher prevalence of aortic valve calcification compared to the general population.^10^ The primary treatments for advanced AS in SSc include surgical aortic valve replacement (SAVR) and transcatheter aortic valve replacement (TAVR). While SAVR involves open-heart surgery, TAVR is a less invasive procedure that can be more suitable for patients with comorbidities.^9,11–13^

Preliminary studies of TAVR in SSc patients indicate promising outcomes, with high success rates and improved functional status. However, SSc patients are at increased risk for infections and thromboembolic events, necessitating careful postoperative monitoring. Comparatively, TAVR shows lower rates of readmission and shorter hospital stays than SAVR making it a preferred option for high-risk patients .^9,10,14–16^

In this retrospective chart review of all SSc patients who underwent a TAVR procedure at our institution over an 11-year period, we aim to provide an understanding of the short- and long-term outcomes and the safety of this procedure.

## METHODS

Following Institutional Review Board approval, patients at the University of Pennsylvania between 2012 and 2023 were identified using ICD-9 and ICD-10 diagnosis codes for systemic sclerosis (SSc) and CPT codes for transcatheter aortic valve replacement (TAVR). Patients included in this study were required to fulfill the 2013 ACR/EULAR classification criteria for SSc.^17^ This case series adheres to the CABARET guidelines for reporting case series, as recommended by the EQUATOR network.

Only those who were deemed appropriate TAVR candidates by the institution’s multidisciplinary structural heart team were included, based on evaluation of their clinical status, comorbidities, anatomical suitability, and surgical risk. Patients who underwent surgical aortic valve replacement (SAVR) instead of TAVR, who did not meet formal SSc classification criteria, or who had incomplete clinical records were excluded.

A retrospective chart review was performed to extract demographic information, serological data, and clinical manifestations in order to further classify the type of SSc and assess disease severity. Preoperative data included risk scores (EuroSCORE II and TAVR in-hospital mortality score), left ventricular function, and detailed structural assessments of the aortic valve. Procedural characteristics such as valve type, access approach, and perioperative interventions were recorded. Outcomes included both short-term (up to 30 days postoperatively) and long-term follow-up data. These included echocardiographic parameters, 30-day readmission rates, perioperative complications, valve performance, and all-cause mortality. Long-term outcomes were assessed up to the last recorded clinical follow-up visit.

### Statistical Analysis

Descriptive statistics were used to summarise patient demographics, clinical characteristics, and outcomes. Categorical variables are presented as frequencies and percentages. Continuous variables are reported as median values with corresponding minimum and maximum ranges. Given the small sample size (n=14), no inferential or multivariable statistical analyses were performed. This study was not powered for hypothesis testing, and the results are intended to be exploratory and descriptive in nature.

### Comprehensive Search Strategy

A targeted literature search was conducted using PubMed, Scopus, and Web of Science to identify relevant studies on systemic sclerosis (SSc), valvular heart disease, and outcomes of transcatheter aortic valve replacement (TAVR). Search terms included “systemic sclerosis,” “scleroderma,” “aortic stenosis,” “TAVR,” “transcatheter aortic valve implantation,” and “valvular heart disease.” Only English-language, peer-reviewed studies involving human subjects were included.

## RESULTS

### Demographics

Our research during the period of 2012–2023 identified fourteen SSc patients who underwent a TAVR procedure. Of them ten were female and twelve were described as Caucasians while one was Hispanic and one was an African American. The median age at diagnosis of SSc was 58.0 (46–79) years while the median age at the time of the TAVR procedure was 77.0 (61–87). Thirteen patients had limited cutaneous SSc (lcSSc) and one had diffuse cutaneous SSc (dcSSc). Thirteen patients were positive for antinuclear antibody (ANA) with a median titer of 1:1280, six had a speckled pattern, five had a centromere pattern, one homogeneous, one nucleolar and one diffuse. Two patients were positive for SSA, Scl-70 and RNA Polymerase III antibody. With regards to SSc-related organ involvement, renal involvement in one, lung involvement in four, pulmonary arterial hypertension (PAH) in four, GI manifestations in thirteen, musculoskeletal involvement in five and Raynaud’s in fourteen.

Non-SSc related comorbidities prior to the TAVR included eleven patients with hypertension, eight patients with hyperlipidemia, four patients with cancer (2 bladder, 1 laryngeal, 1 esophageal), three patients with conduction abnormalities (1 atrial fibrillation, 2 AV block requiring a pacer), three patients with peripheral arterial disease (PAD), two patients with chronic obstructive pulmonary disease (COPD), two with chronic kidney disease (CKD), one patient with kidney transplant, one patient with autoimmune hepatitis (**Table 1**).

**Table 1.** Baseline demographics and systemic sclerosis characteristics (N = 14).

**Category**	**Variable**	**Value**
**Demographics**	Age at SSc diagnosis, yrs (Median (Min–Max)	58 (46–79)
	Age at TAVR, yrs (Median (Min–Max)	77.0 (61–87)
	Disease duration, yrs	13.7
	Female sex, n (%)	10 (71)
	Race/ethnicity, n (%)	Caucasian 12 (86); Hispanic 1 (7); African American 1 (7)
SSc subtype	Limited cutaneous, n (%)	13 (93)
	Diffuse, n (%)	1 (7)
**Auto-antibodies**	ANA-positive, n (%) / median titer	13 (93) / 1: 1280
	- Centromere	5 (36)
	- Speckled	6 (43)
	- Homogeneous	1 (7)
	- Nucleolar	1 (7)
	SSA / Scl-70 / RNA-pol III, n (%)	2 / 2 / 2 (each 14)
**SSc organ involvement**	Raynaud’s phenomenon	14 (100)
	Gastro-intestinal	13 (93)
	Interstitial lung disease	4 (29)
	Pulmonary arterial hypertension	4 (29)
	Musculoskeletal	5 (36)
	Renal involvement	1 (7)
**Non-SSc comorbidities**	Hypertension	11 (79)
	Hyperlipidaemia	8 (57)
	Cancer (bladder 2, laryngeal 1, esophageal 1)	4 (29)
	Conduction abnormality	3 (21)
	Peripheral arterial disease	3 (21)
	COPD / CKD	2 (14) each
	Kidney transplant / Auto-immune hepatitis	1 (7) each

### Pre-Operative Data and Imaging Findings

Pre-operatively seven patients were classified under New York Heart Association Functional Classification (NYHA) II and seven patients under NYHA III. The median body mass index (BMI) pre-operatively was 24.6 (17.2–48.1). Data collected from blood investigations showed the median hemoglobin was 10.9 (9.0–14.6) g/dL, the median albumin was 4.0 (3.7–4.7) g/dL, the median creatinine was 1.04 (0.61–1.54) mg/dL, and the median C-reactive protein was 0.7 (0.1–0.9) mg/dL. The EuroSCORE II (defined as the European system for cardiac operative risk evaluation which predicts risk of in-hospital mortality after cardiac surgery (which includes age, sex, insulin depended diabetes, chronic pulmonary dysfunction, neurological or musculoskeletal dysfunction creatinine clearance, critical pre-op state, NYHA class, Canadian Cardiovascular society angina grade, extracardiac arteriopathy, previous cardiac surgery, active endocarditis, left ventricular function, recent MI, pulmonary artery pressure, procedural urgency, extent of procedure need for thoracic aorta surgery) was calculated for each patient to estimate the risk of death after cardiac surgery with a median score for our population being 3.2 (1.0–7.8)%.

Similarly, the median TAVR in-hospital mortality risk score (which includes age, sex, race, GFR, dialysis, NYHA IV, severe chronic lung disease, acuity of the procedure, past cardiac arrest, or cardiogenic shock, pre-procedure inotropes and need for mechanical assistive device) for our population was 2.75 (1.7–4.6)%. None of our patients exhibited a hostile chest (compromised chest wall, such that SAVR was not possible) based on pre-operative surgical data collected, and none of our patients exhibited a porcelain aorta (densely calcified aorta beyond the aortic valve) which could make a TAVR procedure difficult. On the preoperative echocardiography, the calculated median aortic valve area was 0.75 (0.53–0.95) cm^2^, the median gradient across the aortic valve was 40.0 (31.0–85.0) mmHg, the median left ventricular ejection fraction was 70.0 (55–79)% and the median pulmonary artery pressure 25.5 (20.0–93.0) mmHg. Also, eleven patients had diastolic dysfunction, ten had aortic regurgitation, fourteen had mitral regurgitation, twelve had tricuspid regurgitation, and only three had right ventricular dysfunction. Additionally, pre-operative computer tomography angiography to evaluate the topography for the valve replacement revealed the following data in our population. The median aortic valve annulus was 25.0 (21.0–32.0) mm, and the median aortic valve sinus was 33.0 (26.0–38.0) mm. We observed that all patients had calcified aortic valves, three also had calcified outflow tracks and seven had calcified mitral valves. Based on the severity of calcification and stenosis three were classified mild, eight moderate and three as severe (**Table 2**).

**Table 2.** Pre-operative, imaging and procedural data.

**Category**	**Parameter**	**Value**
**Pre-operative status**	NYHA class II / III, n (%)	7 (50) / 7 (50)
	BMI, kg /m^2^ (Median (Min–Max)	24.6 (17.2–41.8)
	EuroSCORE II, %	3.2 (1.0–7.8)
	TAVR in-hospital mortality score, %	2.75 (1.7–4.6)
	Hemoglobin, g dL^−1^	10.9 (9.0–14.6)
	Creatinine, mg dL^−1^	1.04 (0.61–1.54)
	C-reactive protein, mg dL^−1^	0.7 (0.1–0.9)
**Echocardiography**	Aortic valve area, cm^2^	0.75 (0.53–0.95)
	Median AV gradient, mmHg	40.0 (31.0–85.0)
	LVEF, %	70.0 (55.0–79.0)
	Pulmonary artery pressure, mmHg	25.5 (20.0–93.0)
	Diastolic dysfunction, n (%)	11 (79)
	Mitral / Tricuspid / Aortic regurgitation, n (%)	14 (100) / 12 (86) / 10 (71)
	RV dysfunction, n (%)	3 (21)
**CT angiography**	Annulus diameter, mm	25.0 (21.0–32.0)
	Sinus diameter, mm	33.0 (26.0–38.0)
	Valve calcification (mild / moderate / severe)	3 / 8 / 3
**Procedural details**	Femoral access, n (%)	14 (100)
	Valve type (Sapien / Evolute / CoreValve)	9 / 3 / 2
	Adjunct PCI / SMA stent, n	2 / 1
	Length of stay, days	4.0 ± 1.2

### Perioperative

All fourteen patients underwent a femoral approach for their TAVR procedure of which nine received a Sapien bioprosthetic valve, three received an Evolute and two received a Corevalve. Two patients had to have a pre-operative coronary stent placed while one patient had to have a stent of the superior mesenteric artery. The post-operative period was uneventful except for one patient who had post-operative volume overload that required treatment with increased doses of diuretics, and one patient who had hyperkalemia, both patients required an admission withing 30 days of the TAVR procedure. The mean hospital stay was 4.0 + 1.2 days. Five patients had an immediate postoperative cardiac conduction disturbance that self-resolved, with two patients having a left bundle branch block (LBBB), two having a 1^st^ degree AV block and one patient having SVT. Echo done within 24 hours of the TAVR procedure revealed good placement in all patients with seven patients having mild paravalvular regurgitation while in one patient it was moderate. Three patients had a postoperative infection not related to the procedure and were treated adequately. None of the patients had postoperative stroke symptoms, though all patients were treated with antiplatelet therapy after the procedure (**Table 2**).

### Postoperative Outcomes

Two patients had postoperative readmission within 30 days, one patient with hyperkalemia and the other with shortness of breath. The patient with shortness of breath developed moderate peri-valvular regurgitation at the TAVR site and a balloon valvuloplasty was attempted to correct it. Both patients were managed and discharged after two days with a NYHA class II at discharge. Later in the course, all patients were followed up to a median duration of 34.0 (9.0–94.0) months. We found that only one patient underwent the removal of the TAVR and a revision SAVR was performed at 39 months post-TAVR. This was the same patient who had a 30-day readmission for shortness of breath and underwent a balloon valvuloplasty.

Altogether, the latest echocardiogram after the surgery showed well-placed bioprosthetic valves in all patients except for a few. Five patients had paravalvular regurgitation (four mild and one moderate) consistent with results of the immediate post-operative period, and the median gradient across the TAVR was 11.5 (6.0–31.0) mmHg.

At the final clinic follow up, eight patients had a paravalvular leak post-procedure, which was mild in seven and moderate in one case. By long-term follow-up, only five patients continued to have a detectable paravalvular leak, suggesting possible resolution over time in some cases. Eight patients had an NYHA class I and six patients had an NYHA class II at last follow-up. Five out of the fourteen patients died during the follow-up period. The causes of death were variable and included cardiac arrest in one, acute myelogenous leukaemia (AML) and acute renal failure (ARF) in one, cerebral vascular accident with subsequent aspiration and respiratory failure in one, septic shock leading to cardiac arrest in one, and suicide in one patient (**Table 3**).

**Table 3.** Early and long-term clinical outcomes.

**Category**	**Outcome**	**Value**
**Early outcomes**	Procedural success, n (%)	14 (100)
	In-hospital complications	Volume overload 1; conduction disturbance 5; infection 3; stroke 0
	30-day readmissions, n (%)	2 (14)
	30-day mortality, n (%)	0
**Long-term outcomes**	Median follow-up, months	34.0 (9.0–94.0)
	NYHA I / II at last follow-up, n	8 / 6
	Paravalvular leak – mild/moderate	4 / 1
	Median TAVR gradient, mmHg	11.5 (6.0–31.0)
	Re-intervention (TAVR → SAVR), n (%)	1 (7) @ 39 months
	Overall deaths, n (%)	5 (36)
	– Cardiac arrest	1
	– AML + acute renal failure	1
	– CVA → respiratory failure	1
	– Septic shock	1
	– Suicide	1

## COMPREHENSIVE LITERATURE REVIEW

Valvular heart disease is a common complication in various rheumatic diseases, including Rheumatoid Arthritis (RA) and Systemic Lupus Erythematosus (SLE).^18–23^ Research shows that patients with these conditions often develop mitral regurgitation (MR) and aortic insufficiency (AI) due to thickening and fibrosis of these heart valves.^18–23^

On autopsy, nearly half of SLE patients were found to have valvular lesions,^24^ while Roldan et al.25 demonstrated that 61% of SLE patients had some valvular abnormality when assessed with transoesophageal echocardiography. Patients with SLE were 3.5 and 3 times more likely to develop MR and AR respectively, compared to the general population.^25–28^

In a study by Kurmann et al.,^29^ the risk of having moderate to severe VHD was found to be four-fold higher in SSc patients. Outcomes of a case-control study from Spain^5^ are very similar to those of a newer study from France,^30^ indicating that nearly half of the SSc patients had mild MR. Nonetheless, mitral stenosis (MS) and AI were found in 2% and 18% of patients, respectively, according to the latter study.

Colaci et al.^31^ reported that the most common form of valvular abnormality in patients with SSc was regurgitation, predominantly in the mitral and tricuspid valves, while the aortic valve accounted for 25% of the valvular abnormalities. Aortic involvement in scleroderma manifests as aortic valve stenosis (AS),^9^ with increased aortic stiffness and a higher prevalence of aortic valve calcification compared to the general population.^10^

The primary treatments for advanced aortic valve stenosis are SAVR and TAVR; however, scleroderma patients often present with comorbidities such as pulmonary hypertension, restrictive lung disease, and esophageal dysmotility, which can complicate surgery and increase perioperative risks. Furthermore, the high prevalence of pulmonary complications in scleroderma patients can make anesthesia and postoperative care particularly challenging.

TAVR, a less invasive alternative to SAVR, involves the percutaneous insertion of a bioprosthetic valve via a catheter. TAVR has emerged as a feasible and effective option for high-risk surgical patients, including those with scleroderma. The choice between SAVR and TAVR depends on various factors, including the patient’s surgical risk, anatomical considerations, and the presence of comorbid conditions. Studies suggest that TAVR is often preferred due to its minimally invasive nature, which is crucial for patients with significant comorbidities,^14,15^ though the decision must be individualised, considering the specific risks and benefits for each patient.^16^

Several studies have compared outcomes of TAVR and surgical aortic valve replacement (SAVR) in patients with connective tissue diseases, including systemic sclerosis. TAVR has been shown to be associated with lower in-hospital morbidity, shorter length of stay, and fewer perioperative complications when compared to SAVR, particularly in high-risk patients with pulmonary or esophageal involvement.

Scleroderma patients undergoing TAVR are at a higher risk of certain complications, particularly infectious ones. Rudasill et al.^32^ found that CTD patients had higher odds of readmission for postoperative infection and septicemia compared to non-CTD patients. This heightened risk is attributed to the immunosuppressive therapies commonly used in scleroderma management, which can increase susceptibility to infections.^32^

Additionally, it has been reported that the stroke rate is increased among individuals with CTD undergoing TAVR, indicating a need for careful neurological monitoring post-procedure.^16,32^ The propensity for thromboembolic events in scleroderma patients undergoing TAVR necessitates rigorous anticoagulation.^33^

The readmission rate for scleroderma patients undergoing TAVR is comparable to that of non-CTD patients. Studies have shown similar 30-day readmission rates between the two groups, indicating that TAVR does not lead to higher immediate postoperative readmissions in scleroderma patients.^10^ However, the risk of long-term complications such as infections remains higher, necessitating careful post-procedural monitoring,^32^ and comparative studies have highlighted the advantages of TAVR over SAVR in terms of shorter hospital stays and quicker recovery times.^33^

## DISCUSSION

In this retrospective case series, we examined 14 patients with systemic sclerosis (SSc) who underwent transcatheter aortic valve replacement (TAVR) over an 11-year period. We found that TAVR was well-tolerated, with 100% procedural success and no intraprocedural mortality. Most patients showed improved or stable functional status at long-term follow-up, and only one patient required conversion to surgical valve replacement. Postoperative complications were generally mild and manageable, with no procedure-related deaths. These findings support the safety and feasibility of TAVR in this complex patient population.

Our patients were predominantly older Caucasian females with limited cutaneous SSc, diagnosed in their 60s and undergoing TAVR roughly a decade later. The predominance of limited cutaneous subtype and the high prevalence of comorbidities such as hypertension, lung disease, and conduction abnormalities underscore the clinical complexity of this population. Preoperatively, half of the cohort were NYHA class II and half were NYHA class III. At follow-up, the majority had improved to NYHA class I, supporting a favorable functional response. Notably, all patients had some degree of valve calcification, and over half had mitral involvement in addition to severe aortic stenosis. Despite this, procedural success was 100%, and the femoral approach was used in all cases.

Conduction disturbances occurred in several patients post-TAVR, but all resolved without need for permanent pacing. Similarly, paravalvular leak was common, though mostly mild and stable over time. Only one patient required re-intervention with SAVR, more than three years after the index procedure, underscoring the importance of close monitoring.

While five patients died during follow-up, causes were varied and unrelated to the procedure. These findings reflect the chronic, multisystem burden of SSc rather than TAVR-related complications.

Our findings are consistent with prior literature suggesting that TAVR is a safe and effective option for high-risk patients with SSc. Although the study is limited by its retrospective design and small sample size, it adds valuable data to a relatively understudied patient population. Larger, multicenter studies are needed to validate these results and guide future management strategies in this complex cohort.

## CONCLUSIONS AND FUTURE PERSPECTIVES

Our work in addition to the work of others comes to improve our understanding on how to better manage elderly SSc patients with advanced aortic stenosis. Current data on TAVR in SSc is limited though by adding the information of this study with that of pre-existing studies we can conclude that TAVR is a safe treatment option for SSc patients who have advanced aortic valve stenosis. Our patients are elderly and have multiple SSc and non-SSc related comorbidities making SAVR a less desirable option. Further studies are warranted to solidify these findings and optimise the management of aortic valve stenosis in scleroderma patients. Future research should focus on multicenter prospective cohorts and explore patient-specific predictors of TAVR success in this unique population.

## OPEN DATA SHARING NOTE

The datasets analysed during the current study are available from the corresponding author upon reasonable request. De-identified data will be made publicly available in an institutional or open-access repository upon acceptance for publication.
